# Multigenerational family coaggregation study of obsessive-compulsive disorder and cardiometabolic disorders

**DOI:** 10.1136/bmjment-2024-301323

**Published:** 2025-01-19

**Authors:** Anna Holmberg, Josep Pol-Fuster, Ralf Kuja-Halkola, Henrik Larsson, Paul Lichtenstein, Zheng Chang, Brian M D’Onofrio, Isabell Brikell, Anna Sidorchuk, Kayoko Isomura, James J Crowley, Lina Martinsson, Christian Rück, David Mataix-Cols, Lorena Fernández de la Cruz

**Affiliations:** 1 Centre for Psychiatry Research, Department of Clinical Neuroscience, Karolinska Institutet & Stockholm Health Care Services, Region Stockholm, Stockholm, Sweden; 2 Department of Medical Epidemiology and Biostatistics, Karolinska Institutet, Stockholm, Sweden; 3 School of Medical Sciences, Örebro University, Örebro, Sweden; 4 Department of Psychological and Brain Sciences, Indiana University, Bloomington, Indiana, USA; 5 Department of Global Public Health and Primary Care, University of Bergen, Bergen, Norway; 6 Department of Biomedicine, Aarhus University, Aarhus, Denmark; 7 Department of Genetics, University of North Carolina at Chapel Hill, Chapel Hill, North Carolina, USA; 8 Department of Clinical Sciences, Lund University, Lund, Sweden

**Keywords:** PSYCHIATRY

## Abstract

**ABSTRACT:**

**Background:**

Obsessive-compulsive disorder (OCD) is associated with an increased risk of morbidity and mortality due to cardiometabolic disorders. Whether this association is driven by familial factors is unknown. This population-based family study explored the familial co-aggregation of OCD and cardiometabolic disorders.

**Methods:**

We identified 6 049 717 individuals born in Sweden between 1950 and 2008, including 50 212 individuals with OCD, and followed them up to 2020. These individuals were linked to their mothers, fathers, full siblings, maternal and paternal half siblings, aunts, uncles and cousins. We estimated the risk of cardiovascular diseases (CVD) and metabolic disorders (including obesity, type 2 diabetes and hyperlipidaemia), comparing the relatives of probands with and without OCD. Cox proportional hazards regression models, incorporating time-varying exposures, estimated HRs.

**Results:**

OCD was associated with an increased risk of CVD (HR 1.47; 95% CI 1.43 to 1.51), obesity (HR 1.69; 95% CI 1.63 to 1.74), type 2 diabetes (HR 2.01; 95% CI 1.90 to 2.12) and hyperlipidaemia (HR 1.42; 95% CI 1.33 to 1.52). The relatives of probands with OCD exhibited small increased risks of CVD (HRs from 1.01 to 1.11) and obesity (HRs from 1.03 to 1.20). Slightly increased risks for type 2 diabetes were observed in mothers (HR 1.11; 95% CI 1.07 to 1.15) and full siblings (HR 1.12; 95% CI 1.05 to 1.20), while for hyperlipidaemia it was only observed in mothers (HR 1.06; 95% CI 1.02 to 1.10).

**Conclusions:**

Our results do not support a major contribution of familial factors to the association between OCD and cardiometabolic disorders, suggesting a more prominent role of unique environmental factors.

**WHAT IS ALREADY KNOWN ON THIS TOPIC**

- Obsessive-compulsive disorder (OCD) is associated with an increased risk of morbidity and mortality due to cardiometabolic disorders. However, the mechanisms driving this association remain largely unexplored.

**WHAT THIS STUDY ADDS**

- This study found that familial factors (i.e., shared genetic and environmental factors) do not strongly contribute to the association between OCD and cardiometabolic disorders, suggesting a likely influence of unique environmental factors, such as unhealthy lifestyle habits or medication use.

**HOW THIS STUDY MIGHT AFFECT RESEARCH, PRACTICE OR POLICY**

- Further study of the unique environmental factors that might mediate the association between OCD and cardiometabolic outcomes is needed. Strategies to promote healthy lifestyles in individuals with OCD should be considered.

## Introduction

 Obsessive-compulsive disorder (OCD) is a moderately heritable psychiatric disorder^1 2^ associated with an increased risk of mortality due to both natural and unnatural causes of death.^3 4^ The evidence of an association between OCD and morbidity and mortality due to endocrine, metabolic and circulatory system diseases, including cardiovascular diseases (CVD), is robust,^4^,^7^ but the mechanisms underlying these associations are poorly understood.

A number of mental disorders have been shown to share genetic risk factors with cardiometabolic conditions. For example, genetic risk for schizophrenia is associated with cardiac structure and function^8^ and an increased risk of heart failure.^9^ Further, an overlap has been found between genes associated with both cardiovascular and metabolic disorders and depression and bipolar disorder.^10 11^ Thus, it is plausible to think that there may also be a genetic overlap between OCD and cardiometabolic disorders. However, the evidence supporting this hypothesis is currently sparse.

A few population-based studies have found minimal or no attenuation of the magnitude of the associations between OCD and cardiometabolic outcomes when OCD-affected individuals were compared with their unaffected full siblings,^4 6 7^ suggesting a small contribution of shared familial factors. In the largest OCD genome-wide association study (GWAS) to date,^12^ including over 50 000 individuals with OCD and over 2 million controls, there were no significant genetic correlations with any cardiovascular phenotypes. Nevertheless, these studies should not be considered to provide definitive evidence to rule out a genetic contribution. Residual genetic confounding is still possible in sibling comparisons, given that full siblings only share about 50% of their genetic variance, and GWAS results primarily reflect common genetic variants. Thus, further research using complementary study designs is needed for a more nuanced understanding of the association between OCD and cardiovascular outcomes.

In this Swedish population-based study including more than 6 million individuals, we explored whether OCD and cardiometabolic disorders coaggregate in the same families. If familial factors (ie, genetic and/or environmental factors shared by relatives) played a substantial role in such association, we would expect that the biological relatives of individuals with OCD would have higher risks of cardiometabolic disorders, compared with relatives of individuals without OCD, and that the strength of the associations would increase alongside genetic relatedness. Specifically, if the associations were strongest among first-degree relatives, followed in decreasing order by second-degree and third-degree relatives, this would be suggestive of shared genetic effects. In the absence of such gradient, a shared genetic explanation for the co-occurrence of OCD and cardiometabolic disorders would be less plausible.

## Methods

Because the study was register based and individuals were not identifiable at any time, the requirement for informed consent was waived.

### Data sources

We used the unique Swedish personal identification number to link several health and administrative registers. The Total Population Register, which includes information on all Swedish residents, as well as information on emigration and immigration from and to Sweden since 1961 and 1969, respectively, facilitated the identification of the study cohort and the acquisition of migration data. The Cause of Death Register, covering dates and causes of all deaths since 1961, was used to obtain information on deaths. The Multi-Generation Register, with information about kinship of each person born from 1932 and for those registered in Sweden after 1960, was used to identify biological kinships. The National Patient Register (NPR), covering all inpatient hospital admissions since 1969 and outpatient specialist care since 2001, was used to obtain data on clinical diagnoses. Diagnoses were based on the Swedish version of the International Classification of Diseases (ICD), eighth (ICD-8; 1969–1986), ninth (ICD-9; 1987–1996) and tenth (ICD-10; 1997 and onwards) revisions.

### Study cohorts

The study population consisted of all individuals born in Sweden between 1 January 1950 and 31 December 2008 who had information on both biological parents. Individuals who died at birth and had no follow-up data, and individuals who emigrated, died or had the outcomes of interest before 1973 (the year from which the NPR is comprehensive for psychiatric diagnoses)^13^ were excluded.

We used individuals in this cohort (probands) to identify proband-relative pairs and construct eight subcohorts of relatives with different degrees of relatedness: mothers, fathers, full siblings (ie, siblings sharing both parents), maternal half siblings (ie, siblings with the same mother but different father), paternal half siblings (ie, siblings with the same father but different mother), aunts, uncles and cousins (ie, individuals that share two grandparents) (online supplemental figure 1). The subcohorts of full siblings, maternal and paternal half siblings, and cousins included only proband-relative pairs within the study population. In contrast, the subcohorts of parents and aunts/uncles could include relatives from outside the study population. To mitigate generational differences in these subcohorts, we excluded from the analyses proband-relative pairs with relatives born before 1930 or after 1990, as well as those whose relatives died, emigrated or had the outcomes of interest before 1973. Probands in the cohort and relatives in the subcohorts were followed from birth or from 1 January 1973, whichever came last, until the date of the outcome diagnosis, emigration, death or 31 December 2020 (end of the study period), whichever came first.

### Exposure

In accordance with previous OCD register-based studies,^4 7^ we identified the first instance of an OCD diagnosis in the NPR (ICD-8: 300.3; ICD-9: 300D; ICD-10: F42) if recorded after the age of 6 (to avoid misclassification of cases). Furthermore, in within-individual analysis, given that the date for OCD diagnosis in the registers is a poor representation of the actual onset of the disorder, individuals with OCD were considered unexposed before age 6 and exposed either from age 6 or the year 1973, whichever came last. In the subcohorts of relatives, exposure was defined as an OCD diagnosis in the proband. Relatives were considered unexposed prior to the proband’s OCD diagnosis (either age 6 or 1973) and exposed thereafter. The codes for OCD in the NPR have excellent inter-rater reliability and moderate to excellent validity.^14^

### Outcome

From the NPR, we identified the first record of the following four outcome variables: (1) a broad group of CVDs (including acute rheumatic fever, chronic rheumatic heart diseases, hypertensive diseases, ischaemic heart diseases, pulmonary heart disease, other forms of heart disease, cerebrovascular diseases, atherosclerosis, Raynaud syndrome, arterial embolism and thrombosis), (2) obesity, (3) type 2 diabetes and (4) hyperlipidaemia (see ICD codes in online supplemental table 1).

### Statistical analysis

A coaggregation study involves analysing the co-occurrence of two traits in probands and their relatives and comparing the strength of the association between relatives of exposed and unexposed probands. If the two traits are found to co-occur, it suggests that familial factors are important contributors to the association between them. Given the differences in genetic and environmental factors shared among different types of relatives, comparing clusters of relatives with different degree of relatedness allows making inferences about the specific contribution of familial factors.

In the whole cohort, we first explored the risk of CVD and metabolic disorders in individuals with OCD, compared with individuals without OCD. To that end, we fitted Cox regression models using age as underlying timescale and with time-varying exposure to calculate HRs and 95% CIs. These analyses were adjusted for sex and birth year (categorised in 10-year increments).

To evaluate the familial coaggregation of OCD in the probands with each separate outcome variable in the relatives, we fitted a series of Cox regression models using age as underlying timescale and with time-varying exposure in the cohorts of relatives. These models compared the risk of cardiometabolic outcomes in relatives of individuals exposed to OCD, compared with relatives of individuals without OCD. The models were adjusted for birth year (categorised in 10-year increments) and sex of both the proband and the relative in all cohorts, except for analyses in mothers, fathers, aunts and uncles, which were not adjusted for the relative’s sex (model 1). In a second model, we explored whether familial coaggregation was better explained by the direct effect of OCD within an individual by additionally adjusting for OCD in the relatives (model 2).

Cluster robust SEs were applied in all analyses to address familial clustering. Data analyses were conducted between 1 October 2023 and 1 May 2024 in SAS (V.9.4; SAS Institute) and R using survival package.

## Results

The cohort included 6 049 717 individuals, of whom 50 212 (0.83%) were diagnosed with OCD during the study period. The main characteristics of the OCD and non-OCD cohorts are summarised in table 1. The median age at first OCD diagnosis was 24.6 years (IQR: 16.2). The median age at first diagnosis of the different outcomes was as follows: 46.8 years (IQR: 18.7) for CVD, 34.2 years (IQR: 23.9) for obesity, 46.8 years (IQR: 20.6) for type 2 diabetes and 52.7 years (IQR: 12.6) for hyperlipidaemia. Table 2 shows the relatives identified for each proband in the cohort.

**Table 1:** Cohort descriptive information

Characteristics	OCD, n (%)	No record of OCD, n (%)
Sex*		
Male	21 128 (42.1)	3 086 867 (51.4)
Female	29 084 (57.9)	2 912 638 (48.5)
Birth year*		
1950–1959	3402 (6.8)	993 795 (16.6)
1960–1969	5361 (10.7)	1 071 591 (17.9)
1970–1979	8089 (16.1)	1 017 401 (17)
1980–1989	12 652 (25.2)	981 243 (16.4)
1990–1999	13 596 (27.1)	1 043 104 (17.4)
2000–2008	7112 (14.2)	892 371 (14.9)
Outcomes		
Cardiovascular diseases*	5294 (10.5)	695 602 (11.6)
Obesity*	3624 (7.2)	240 161 (4.0)
Type 2 diabetes*	1374 (2.7)	143 240 (2.4)
Hyperlipidaemia*	827 (1.7)	133 209 (2.2)

* Statistically significant difference at p<0.001 from a Chi-squareχ2 test (categorical) when comparing OCD versus non-recorded OCD cohorts.

OCDobsessive-compulsive disorder

**Table 2:** Number of probands in the study cohort and each cluster of relatives

Total cohort and family clusters	Unique probands	Unique pairs*	Observations†	Excluded
Total cohort	6 049 717		6 049 717	
Mothers	5 206 873	5 206 873	5 206 873	842 844‡
Fathers	4 894 646	4 894 646	4 894 646	1 155 071‡
Full siblings	4 696 684	3 828 829	7 657 658	1 353 033§
Maternal half siblings	807 962	635 220	1 270 440	5 241 755§
Paternal half siblings	915 815	802 194	1 604 388	5 133 902§
Aunts	3 340 959	6 571 481	6 571 481	2 708 758¶
Uncles	3 426 412	6 913 154	6 913 154	2 623 305¶
Cousins	3 913 728	12 601 798	25 203 596	2 135 989**

* Number of unique pairs identified (eg, Ooffspring– Mmother, Ssibling 1–Ssibling 2).

† Number of observations included (ie, all possible combinations of pairs in which members contribute to the analysis with information on exposure and outcome). Each sibling and cousin contributed at least once with data on obsessive-compulsive disorder and cardiometabolic outcomes. Parents, aunts, and uncles contributed only to cardiometabolic outcomes.

‡ Probands whose parents were born before 1930 or after 1990, died, emigrated or had the outcomes of interest before 1973.

§ Probands with no siblings of a certain degree of relatedness identified from the study cohort.

¶ Probands whose uncles/aunts were twins of their parents, born before 1930 or after 1990, died, emigrated, or had the outcomes of interest before 1973.

** Probands with no cousins identified from the study cohort or if parents of cousins were twins.

**Figure 1:**
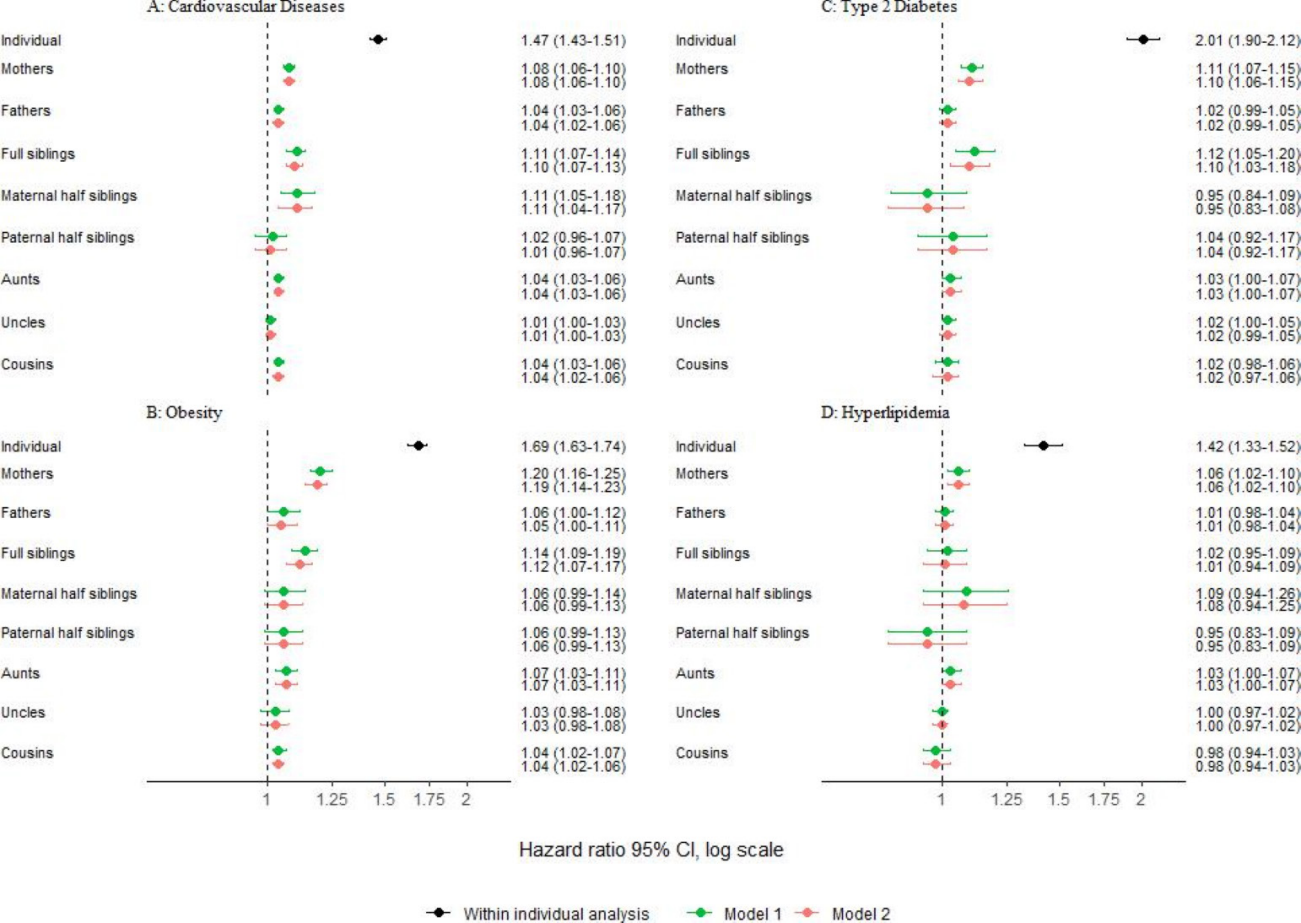
Coaggregation of obsessive-compulsive disorder with cardiovascular diseases (**A**), obesity (**B**), type 2 diabetes (**C**) and hyperlipidaemia (**D**). The within-individual risk models (black) were adjusted for birth year (categorised in 10-year increments) and sex. Model 1 (green) adjusted for birth year (categorised in 10-year increments) and sex of the proband and the relative, when needed. Model 2 (red) additionally adjusted for obsessive-compulsive disorder diagnoses in the relatives.

Individuals with OCD had an increased risk of all four outcomes: CVD (HR 1.47; 95% CI 1.43 to 1.51), obesity (HR 1.69; 95% CI 1.63 to 1.74), type 2 diabetes (HR 2.01; 95% CI 1.90 to 2.12) and hyperlipidaemia (HR 1.42; 95% CI 1.33 to 1.52), compared with unaffected individuals, after adjusting for sex and birth year (figure 1).

Relatives of individuals with OCD showed a small although statistically significant increased risk of CVD, compared with the relatives of individuals without OCD (model 1 in figure 1A). Notably, first-degree relatives exhibited the highest risks, including full siblings (HR 1.11; 95% CI 1.07 to 1.14), mothers (HR 1.08; 95% CI 1.06 to 1.10) and fathers (HR 1.04; 95% CI 1.03 to 1.06). Second-degree relatives displayed slightly smaller risks, compared with first-degree relatives, including paternal half siblings (HR 1.02; 95% CI 0.96 to 1.07), aunts (HR 1.04; 95% CI 1.03 to 1.06) and uncles (HR 1.01; 95% CI 1.00 to 1.03). Risk of CVD in maternal half siblings (HR 1.11; 95% CI 1.05 to 1.18) was comparable to that in the first-degree relatives, although CIs were broader for this cluster. Additionally, cousins displayed a similar risk to that observed in second-degree relatives (HR 1.04; 95% CI 1.03 to 1.06).

Similar patterns were observed for obesity (model 1 in figure 1B). First-degree relatives of individuals with OCD exhibited the highest risks of obesity: mothers (HR 1.20; 95% CI 1.16 to 1.25), fathers (HR 1.06; 95% CI 1.00 to 1.12) and full siblings (HR 1.14; 95% CI 1.09 to 1.19). Second-degree relatives displayed attenuated estimates, although with overlapping CIs, compared with first-degree relatives: maternal half siblings (HR 1.06; 95% CI 0.99 to 1.14), paternal half siblings (HR 1.06; 95% CI 0.99 to 1.13), aunts (HR 1.07; 95% CI 1.03 to 1.11) and uncles (HR 1.03; 95% CI 0.98 to 1.08). The risk in cousins was similar to that observed in second-degree relatives (HR 1.04; 95% CI 1.02 to 1.07).

For type 2 diabetes, no pattern of coaggregation was observed, with most estimates being close to the null and non-significant. Only full siblings (HR 1.12; 95% CI 1.05 to 1.20) and mothers (HR 1.11; 95% CI 1.07 to 1.15) exhibited slightly increased risks (model 1 in figure 1C). Similarly, for hyperlipidaemia, only mothers exhibited a slightly increased risk (HR 1.06; 95% CI 1.02 to 1.10) and no clear pattern of coaggregation was observed (model 1 in figure 1D).

The pattern of results remained overall unchanged throughout all outcomes after adjusting for OCD in the relatives, although with slightly attenuated estimates (model 2 in figure 1).

## Discussion

In line with earlier population-based studies, this study found an increased risk of morbidity due to cardiometabolic conditions in individuals with OCD.^5^,^7^ However, the coaggregation analyses revealed limited evidence for shared familial risk factors between OCD and cardiometabolic disorders. The magnitude of the associations in the relatives was small, especially when compared with the within-individual analyses. Additionally, we did not observe a clear gradient of increased risks with increasing genetic relatedness, with the possible exception of obesity.

Our findings are consistent with previous evidence from sibling-controlled studies which had found that the association between OCD and cardiometabolic conditions appeared to be largely independent of familial confounding.^6 7^ Furthermore, the largest OCD GWAS to date did not find significant genetic correlations between OCD and several cardiovascular and metabolic phenotypes, including myocardial infarction, coronary artery disease, type 2 diabetes, cholesterol and triglycerides.^12^ Thus, while the presence of shared genetic risk factors cannot be fully ruled out, their contribution appears to be small or negligible, particularly for type 2 diabetes and hyperlipidaemia.

Several unique environmental risk factors might mediate the association between OCD and cardiometabolic outcomes.^3 15^ For example, unhealthy lifestyle habits are known risk factors for cardiometabolic disorders and have shown to be common in psychiatric disorders.^16^ OCD has been associated with an increased risk of substance misuse,^17^ smoking^18^ and sleep problems.^19 20^ Additionally, individuals with OCD self-report low levels of physical activity and unhealthy dietary habits.^15^ Regardless of the mechanisms, the promotion of a healthy lifestyle to improve physical health and prevent ill health in individuals with OCD should be considered.^21^ Another risk factor that has been associated with an increased risk of cardiometabolic complications in individuals with other mental disorders, such as schizophrenia, bipolar disorder and depression, is the use of psychotropic drugs.^22^ Weight gain and metabolic effects are common side effects of antipsychotics and some, but not all, antidepressants.^16^ In OCD, the recommended pharmacological treatment includes medication with selective serotonin reuptake inhibitors, which is commonly prescribed in this patient group.^23^ However, the effects of psychotropic medication on cardiometabolic health have barely been studied, and results from observational studies have been inconsistent.^5 7 24^ Further study of the effects of these medications in the cardiometabolic health of individuals with OCD is warranted. Finally, it is known that individuals with mental disorders experience inequalities in the use and provision of healthcare services,^16^ which could lead to delays in the detection and treatment of common diseases, leading to worse outcomes.^3^

### Strengths and limitations

To our knowledge, this study is the first to explore the familial coaggregation of OCD and cardiometabolic conditions in a large population-based cohort with longitudinally collected data. The Multi-Generation Register made it possible to identify different clusters of relatives with different degrees of relatedness. Moreover, the diagnostic codes used to identify individuals with OCD are highly reliable and valid, particularly ICD-10 codes.^14^ The ICD codes for several CVDs and metabolic disorders have also been validated in the NPR, with generally high positive predictive values.^25^

This study is not without limitations. The coverage of the NPR is limited, with outpatient care diagnosis being introduced only from 2001 and no information from primary care.^25^ Additionally, not all individuals with OCD seek help,^26^ and less severe cardiometabolic conditions may also remain undetected, particularly in individuals with mental disorders.^16^ Consequently, the number of individuals with OCD and cardiometabolic disorders in our study cohort may be underestimated. Data on biological paternity in the Multi-Generation Register are self-reported by the mother and can be inaccurate. However, paternal discrepancy has been reported to be low (1.7%).^27^ Additionally, in Sweden, custody of the children after separation of the parents is currently shared in about 30–45% of the cases,^28 29^ which may diminish potential differences in shared environment between maternal and paternal half siblings. Finally, the Schoenfeld residual test did not support the proportional hazards assumption for our data, which is common in very large datasets.^30^ However, the failure to meet this assumption does not change the overall interpretation of our findings.

## Conclusion

The results of this study do not support a major contribution of familial factors to the association between OCD and cardiovascular and metabolic disorders, suggesting a more prominent role of unique environmental factors, such as unhealthy lifestyles or medication use.

## supplementary material

10.1136/bmjment-2024-301323 online supplemental file 1

## Data Availability

Data may be obtained from a third party and are not publicly available.
