# Supplementary material for: Multigenerational family coaggregation study of obsessive-compulsive disorder and cardiometabolic disorders
Source: BMJ Ment Health. 2025 Jan 19;28(1):e301323. doi: 10.1136/bmjment-2024-301323 (PMC11751958; doi:10.1136/bmjment-2024-301323)
Supplement: online supplemental file 1 [file bmjment-28-1-s001.pdf]

SUPPLEMENTARY MATERIAL

Supplementary Figure 1. Overview of the study design.

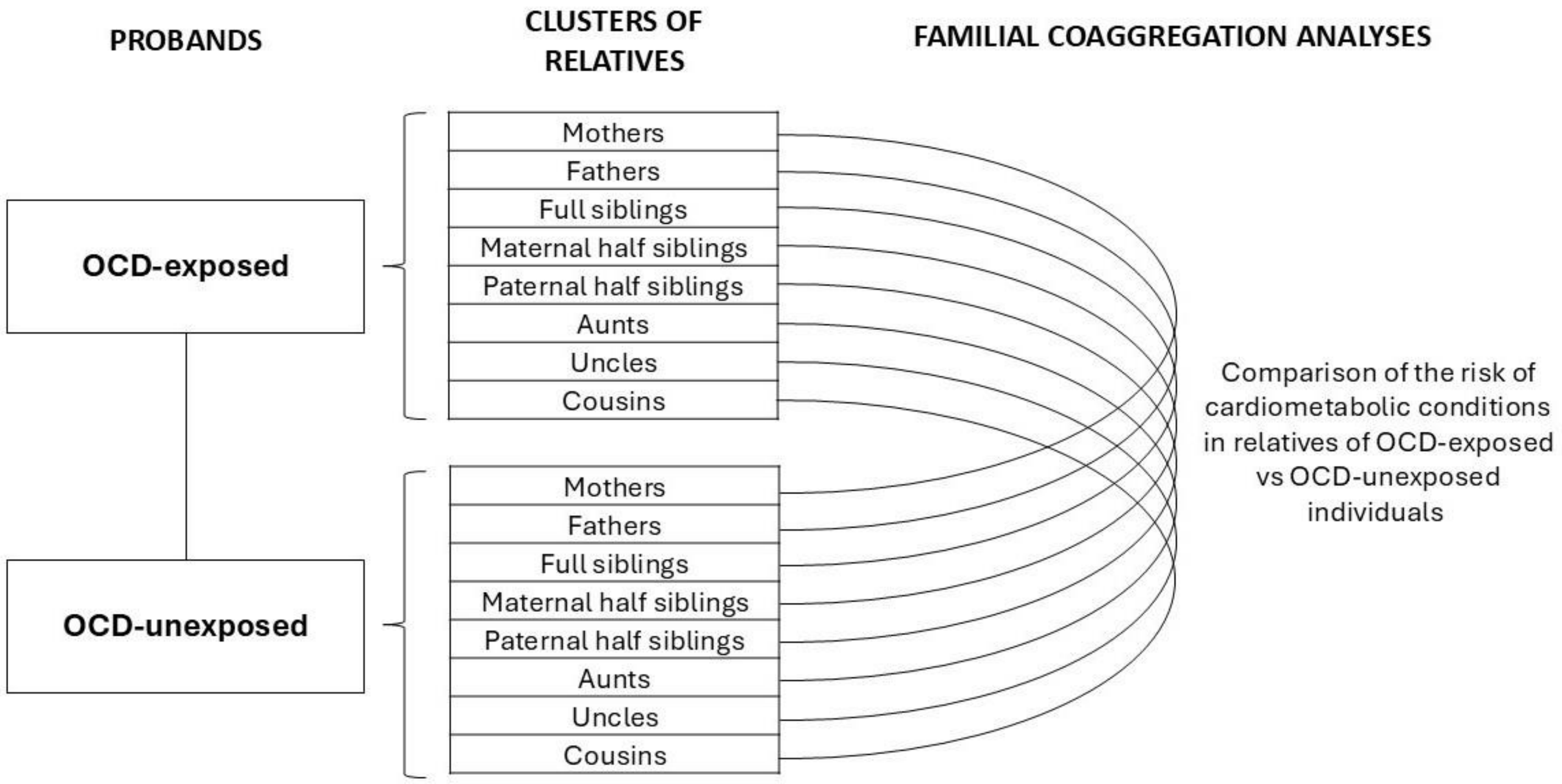

Supplementary Table 1. Swedish International Classification of Diseases 8th (ICD-8), 9th (ICD-9), and 10th (ICD-10) revision diagnostic codes for the cardiometabolic outcomes included in the study.

**Outcomes**	**Swedish ICD-8**	**Swedish ICD-9**	**Swedish ICD-10**
**Cardiovascular diseases**	390-438, 440, 444, 445, 450-453, 458	390-438, 440, 444, 445	I00-I70, I73.0, I74-I75
**Metabolic disorders**			
Obesity	277	278A, 278B	E65, E66
Type 2 diabetes	250	250	E11
Hyperlipidemia	279	272	E78
